# The Cardiovascular and Neurotoxic Effects of the Venoms of Six Bony and Cartilaginous Fish Species

**DOI:** 10.3390/toxins9020067

**Published:** 2017-02-16

**Authors:** Han Han, Kate Baumann, Nicholas R. Casewell, Syed A. Ali, James Dobson, Ivan Koludarov, Jordan Debono, Scott C. Cutmore, Niwanthi W. Rajapakse, Timothy N. W. Jackson, Rob Jones, Wayne C. Hodgson, Bryan G. Fry, Sanjaya Kuruppu

**Affiliations:** 1 Department of Pharmacology, Biomedicine Discovery Institute, Monash University, Clayton, VIC 3800, Australia; hhan24@student.monash.edu (H.H.); dr.syedabidali@gmail.com (S.A.A.); wayne.hodgson@monash.edu (W.C.H.); 2 Venom Evolution Lab, School of Biological Sciences, University of Queensland, St Lucia, QLD 4072, Australia; kate.baumann@uqconnect.edu.au (K.B.); james.dobson@uqconnect.edu.au (Jam.D.); ivan.koludarov@uq.net.au (I.K.); jordan.debono@uqconnect.edu.au (Jor.D.); tnwjackson@gmail.com (T.N.W.J.); 3 Alistair Reid Venom Research Unit, Liverpool School of Tropical Medicine, Pembroke Place, Liverpool L3 5QA, UK; nicholas.casewell@lstmed.ac.uk; 4 HEJ Research Institute of Chemistry, International Centre for Chemical and Biological Sciences (ICCBS), University of Karachi, Karachi 75270, Pakistan; 5 Marine Parisitology Group, School of Biological Sciences, University of Queensland, St Lucia, QLD 4072, Australia; scott.cutmore@uqconnect.edu.au; 6 Baker IDI Heart and Diabetes Institute, 75 Commercial Road, Prahran 3181, Australia; niwanthi.rajapakse@bakeridi.edu.au; 7 Department of Physiology, Biomedicine Discovery Institute, Monash University, Clayton, VIC 3800, Australia; 8 The Aquarium Vet, P.O. Box 2327, Moorabbin, VIC 3189, Australia; rob@theaquariumvet.com.au; 9 Department of Biochemistry & Molecular Biology, Biomedicine Discovery Institute, Monash University, Monash, VIC 3800, Australia

**Keywords:** venom, fish, cardiovascular, neuromuscular, toxin

## Abstract

Fish venoms are often poorly studied, in part due to the difficulty in obtaining, extracting, and storing them. In this study, we characterize the cardiovascular and neurotoxic effects of the venoms from the following six species of fish: the cartilaginous stingrays *Neotrygon kuhlii* and *Himantura toshi*, and the bony fish *Platycephalus fucus*, *Girella tricuspidata*, *Mugil cephalus*, and *Dentex tumifrons*. All venoms (10–100 µg/kg, i.v.), except *G. tricuspidata* and *P. fuscus*, induced a biphasic response on mean arterial pressure (MAP) in the anesthetised rat. *P. fucus* venom exhibited a hypotensive response, while venom from *G. tricuspidata* displayed a single depressor response. All venoms induced cardiovascular collapse at 200 µg/kg, i.v. The in vitro neurotoxic effects of venom were examined using the chick biventer cervicis nerve-muscle (CBCNM) preparation. *N. kuhlii*, *H. toshi*, and *P. fucus* venoms caused concentration-dependent inhibition of indirect twitches in the CBCNM preparation. These three venoms also inhibited responses to exogenous acetylcholine (ACh) and carbachol (CCh), but not potassium chloride (KCl), indicating a post-synaptic mode of action. Venom from *G. tricuspidata*, *M. cephalus*, and *D. tumifrons* had no significant effect on indirect twitches or agonist responses in the CBCNM. Our results demonstrate that envenoming by these species of fish may result in moderate cardiovascular and/or neurotoxic effects. Future studies aimed at identifying the molecules responsible for these effects could uncover potentially novel lead compounds for future pharmaceuticals, in addition to generating new knowledge about the evolutionary relationships between venomous animals.

## 1. Introduction

Venomous marine fish account for nearly two-thirds of the population of venomous vertebrates, and include stingrays, scorpionfish, zebrafish, stonefish, and some species of shark, catfish, and blenny [1,2,3]. Fish venoms are thought to have originated on at least 18 occasions via the process of convergent evolution [2,3,4]. However, while significant research effort has focused on characterising the biological activity of venom from terrestrial animals—particularly snakes—little is known about the composition or biological activity of venom from many species of fish. A main reason that these marine vertebrates remain understudied is the difficulty in obtaining, storing, and extracting venom samples [5]. Nevertheless, marine venoms represent a diverse source of untapped biological compounds which, when considering the utility of toxins isolated from other venomous lineages [6], may be useful as potential research, pharmaceutical, or diagnostic tools. 

The majority of venomous fish are sedentary, slow moving, and live in shallow, protected waters [7]. Their venoms are classified as defensive, and are thought to be used much less frequently than those employed by other venomous animals for predatory purposes [8]. Despite their extensive taxonomic diversity at the organismal level, the venom delivery systems used by the majority of fish are similar, and typically consist of dorsal, pectoral, and/or clitheral spines [2,3]. This is perhaps not surprising, as the venoms are almost exclusively used for defence, and such spines are likely to provide a degree of mechanical protection alongside the chemical defences conferred by their toxic secretions. In addition, the pharmacological activities of fish venoms have been postulated to be similar, despite their numerous independent origins. This is perhaps best evidenced by the clinical effects of envenoming, which are often defined as resulting in considerable pain disproportionate to the wound size, although a diverse array of other symptoms such as itching, erythema, and paralysis have been described, resulting in occasional fatalities due to cardiovascular and neurological systemic effects [9,10,11,12,13].

Surprisingly, little research has focused on the pharmacological or compositional nature of fish venoms. One exception to this is that of the stonefish (*Synanceia* spp.), which due to their medically-important nature have been reasonably well studied in terms of their pharmacology, epidemiology, and clinical aspects of envenoming [14,15,16,17,18,19,20]. In the present study, we address the paucity of information surrounding fish venoms by investigating those secretions from a variety of cartilaginous (the blue-spotted stingray *Neotrygon kuhlii* and the brown whipray *Himantura toshi*, both family Dasyatidae) and bony fish (the dusky flathead *Platycephalus fuscus*, family Platycephalidae; the Luderick Bream *Girella tricuspidata*, family Kyphosidae; the mullet *Mugil cephalus*, family Mugilidae; and the yellowback seabream *Dentex tumifrons*, family Sparidae). 

## 2. Results

### 2.1. Effects of Crude Venoms on the Cardiovascular System 

**Figure 1:**
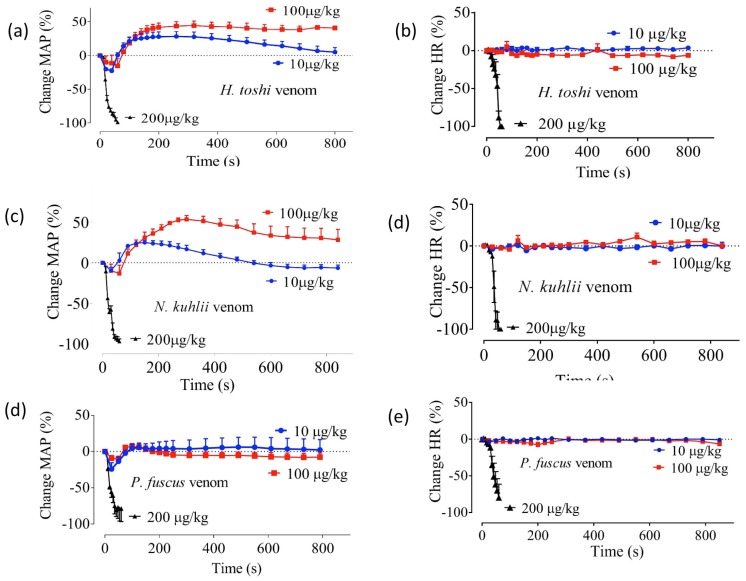
The effect of *H. toshi*, *N. kuhlii*, and *P. fuscus* venoms on mean arterial pressure (MAP; **a**,**c**,**e**, respectively) and heart rate (HR; **b**,**d**,**f**, respectively) of the anaesthetized rat. *N* = 4. Each data point and error bar represents the mean of four experiments and the corresponding SEM, respectively.

Venoms of *H. toshi* and *N. kuhlii* (10–100 μg/kg, i.v.) produced a dose-dependent biphasic effect on mean arterial pressure (MAP), consisting of a depressor response, followed by a sustained pressor response (Figure 1a,c). Venom of *P. fuscus* (10–100 μg/kg, i.v.) only caused a transient depressor response (Figure 1e). All three venoms (10–100 μg/kg, i.v.) had no significant effect on heart rate (Figure 1b,d,f), but caused complete cardiovascular collapse in response to 200 μg/kg, i.v. (Figure 1a–f). 

**Figure 2:**
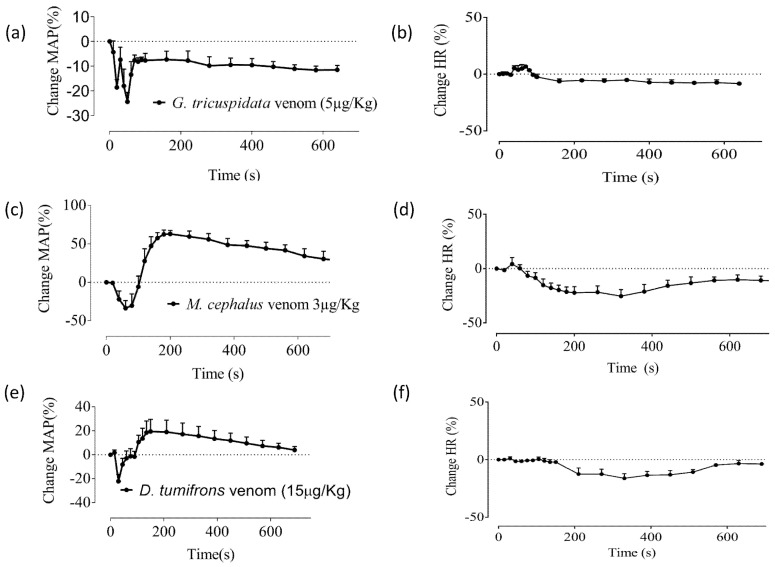
The effect of *G. tricuspidata*, *M. cephalus,* and *D. tumifrons* venoms on mean arterial pressure (MAP; **a**,**c**,**e**, respectively) and heart rate (**b**,**d**,**f**, respectively) of the anaesthetised rat. *N* = 3. Each data point and error bar represents the mean of three experiments and the corresponding SEM.

Interestingly, the venom of *Girella tricuspidata* (5 μg protein/kg, i.v.) produced a biphasic depressor effect consisting of a transient depressor response, followed by a sustained depressor response (Figure 2a), with no significant effect on HR (Figure 2b). *M. cephalus* (3 μg/kg, i.v.) and *D. tumifrons* (15 μg/kg, i.v.) venoms both caused an initial depressor response followed by a pressor response which recovered over time (Figure 2c,e). In contrast to the stingray venoms, both *M. cephalus* and *D. tumifrons* venoms caused small transient decreases in the heart rate of the anaesthetised rat (Figure 2d,f). However, these changes in HR were not significant. The vehicle control group (i.e., saline administration) exhibited no significant effect on either MAP or HR of the anaesthetized rat (data not shown).

### 2.2. Effects of Crude Venoms on the Chick Biventer Cervicis Nerve-Muscle (CBCNM) Preparation 

**Figure 3:**
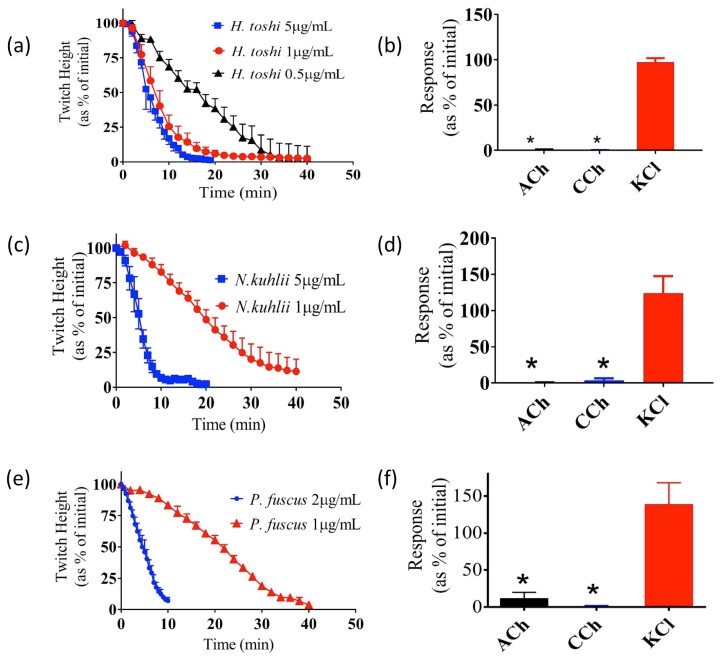
The effect of *H. toshi* (0.5–5 μg/mL), *N. kuhlii* (1–5 μg/mL), and *P. fuscus* (1–2 µg/mL) venom on indirect twitches (**a**,**c**,**e**, respectively), and contractile responses to exogenous acetylcholine (ACh), carbachol (CCh), or KCl (**b**,**d**,**f**, respectively) the CBCNM preparation *N* = 4. * *p* < 0.05, significantly different from the pre-venom baseline, paired *t*-test. Error bars represent the SEM.

**Figure 4:**
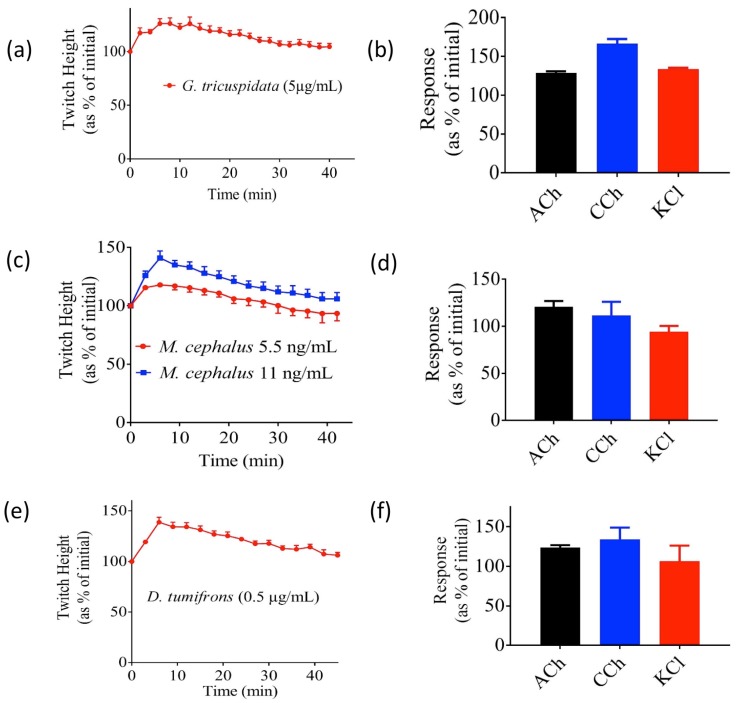
The effect of *G. tricuspidata* (5 μg/mL), *M. cephalus* (5.5–11 ng/mL), and *D. tumifrons* (0.5 µg/mL) venom on indirect twitches (**a**,**c**,**e**, respectively), and contractile responses to exogenous ACh, CCh or KCl (**b**,**d**,**f**, respectively) of the CBCNM preparation. *N* = 4. Error bars represent the SEM.

The venom of the two stingray species—*H. toshi* (0.5–1 µg/mL) and *N. kuhlii* (1–5 µg/mL)—both abolished indirect twitches of the CBCNM preparation in a concentration-dependent manner (Figure 3a,c). Both venoms also significantly inhibited contractile responses to acetylcholine (ACh) and carbachol (CCh), but had no significant effect on responses to KCl (Figure 3b,d). Similarly, venom from the bony fish *P. fuscus* (1–2 µg/mL) was also found to abolish the indirect twitches of the CBCNM preparation in a concentration-dependent manner (Figure 3e), and significantly inhibited the contractile response to ACh and CCh, but not KCl (Figure 3f). The time taken for twitch height to reduce by 50% of initial (i.e., t_50_) in response to 1 µg/mL of *N. kuhlii*, *H. toshi*, and *P. fuscus* venoms was determined. The t_50_ of *H. toshi* venom was significantly less than that of both *N. kuhlii* and *P. fuscus* (*p* < 0.05; one-way ANOVA; *N* = 4, Table 1). In contrast, *G. tricuspidata*, *M. cephalus*, and *D. tumifrons* venoms induced transient increases in indirect twitches of the CBCNM preparation (Figure 4a,c,e), and none of these venoms significantly inhibited contractile responses to ACh, CCh, or KCl (Figure 4b,d,f).

## 3. Discussion

Fish venoms show enormous diversity and complexity of pharmacologically active components [21]. One of the major clinical symptoms observed in humans after fish envenoming is hypotension with marked cardiovascular activity [11]. In this study, we have examined the effect of several fish venoms on the cardiovascular system of anaesthetised rats, paying particular attention to MAP and HR. We have also examined the in vitro neurotoxic effects of these venoms using an avian skeletal muscle preparation. All venoms examined had some effect on the MAP of anesthetised rats, while only the venoms of *N. kuhlii*, *H. toshi*, and *P. fuscus* displayed in vitro neurotoxicity. Interestingly, three of the venoms potentiated twitch height in the skeletal muscle.

In the anesthetised rat, the venoms from *N. kuhlii*, *H. toshi*, and *P. fuscus* induced an initial decrease in MAP at concentrations of 10–100 µg/kg. In the case of *P. fuscus* venom, the MAP returned to baseline. However *N. kuhlii* and *H. toshi* venoms displayed a biphasic response characterised by an initial drop in blood pressure followed by a sustained pressor response. This biphasic response is similar to results observed in previous studies on the Scorpaeniformes *Gymnapistes marmoratus*, *Pterois volitans*, and *Synanceia verrucosa* [22,23,24]. The pressoric cardiovascular responses induced by *G. marmoratus*, *P. volitans*, and *S. verrucosa* venoms were thought to involve an activity dependent on adrenoceptors [22,25,26] and/or to be mediated by non-adrenergic mechanisms [1,27]. Prior research also suggested that the depressor response induced by some fish venoms involved muscarinic receptors and/or nitric oxide synthesis [1,19,27,28,29]. All three venoms—*N. kuhlii*, *H. toshi*, and *P. fuscus*—induced cardiovascular collapse at 200 µg/kg.

Our results indicate that the venom of *G. tricuspidata* induces a sustained drop in MAP at concentrations as low as 5 µg/kg. The venom from *M. cephalus* (3 µg/kg) and *D. tumifrons* (15 µg/kg) also induced a biphasic response similar to that observed after administering venom from *N. kuhlii* and *H. toshi*. The limited supply of venom from these three species precluded us from examining their effects at higher concentrations. However, unlike *N. kuhlii* and *H. toshi* venoms, *M. cephalus* and *D. tumifrons* venoms had a transient negative chronotropic effect on HR, in addition to changes in blood pressure. Previously described chronotropic responses to fish venom have been attributed to different mechanisms, including the activity of chemical mediators in the venom [30], activity on adrenergic and muscarinic receptors [27], or the release of endogenous autacoids in cardiac tissues [22,27,28]. Further studies are required to determine the mechanism(s) responsible for the decrease in HR observed in response to *D. tumifrons* and *M. cephalus* venoms.

Although cardiovascular activity is the most widely reported symptom of fish envenoming, paralysis, muscle spasm, and prolonged weakness have also been noted in envenomed humans [31]. Consequently, we studied the effects of the above-mentioned fish venoms on the skeletal neuromuscular junction, using the chick biventer cervicis nerve-muscle preparation. The venoms from *H. toshi*, *N. kuhlii*, and *P. fuscus* induced a concentration-dependent decrease in indirect twitches of the preparation, with abolition of twitches within 60 min at the tested concentrations. In addition, all three venoms significantly inhibited responses to the nicotinic agonists ACh and CCh, but not to KCl. These results are consistent with the presence of postsynaptic neurotoxins in these venoms. Postsynaptic neurotoxins have been shown to bind with high affinity to nicotinic acetylcholine receptors and competitively antagonise the actions of acetylcholine [32]. Similar findings have been described from the venom of the bony fish *Scatophagus argus* [33]. In contrast, *D. tumifrons*, *G. tricuspidata*, and *M. cephalus* venoms did not abolish indirect twitches of the chick biventer cervicis. Instead, these venoms induced a transient potentiating effect with no significant inhibition of the contractile responses to exogenous agonists.

To the best of our knowledge, this is the first study to examine the neurotoxic and cardiovascular effects of the venoms from these six species of fish. The cardiovascular effects as indicated by changes in MAP were apparent across all of the species tested, even at low concentrations. However, in vitro neurotoxicity was only observed in response to *H. toshi*, *N. kuhlii*, and *P. fuscus* venoms. These results corroborate other findings, suggesting that while most fish venoms are convergently functionally similar due to shared selection pressures for their use as defensive weapons, their effects differ quantitatively due to unique components in different venomous fish lineages [15], in addition to the quantity of the venom delivered [31]. Due to the limited amount of pharmacological data available for fish venoms, our results contribute significantly to our understanding of the functional activity of bony and cartilaginous fish venoms. These results have implications spanning evolutionary theory through to the treatment of envenomed patients.

## 4. Materials and Methods 

### 4.1. Crude Venom

#### 4.1.1. Sample Collection and Storage

Specimens were collected under collection permit QS2013/MAN143 (13/12/13) and animal ethics approval SBS/345/12/ARC (24 December 2015). Spine samples were collected and immediately snap frozen in liquid nitrogen before storage at −80 °C until use. 

##### Chondrichthyes (Cartilaginous Fish)

*Himantura toshi* (Brown Whipray—collected from Moreton Bay, Queensland) and *Neotrygon kuhlii* (Bluespot stingray—collected from Moreton Bay, Queensland)

##### Osteichthyes (Bony Fish)

*Dentex tumifrons* (Yellowbacked Seabream—collected from Moreton Bay, Queensland Australia), *Girella tricuspidata* (Luderick Bream—collected from Moreton Bay, Queensland) *Mugil cephalus* (Flathead Mullet—collected from Moreton Bay, Queensland); and *Platycephalus fuscus* (Flathead Bream—collected from Mornington Peninsula, Victoria and Moreton Bay, Queensland). 

#### 4.1.2. Protein Extraction

The crude venom was extracted from the fish spines and cleaned as previously described [5]. A solution was prepared on ice using 3.7 g EDTA, 5 mL 200 mM PMSF, 10 mL Triton X-100, 1 L purified water. The solution was poured over the spines and placed on a magnetic stirrer overnight (>12 h) at 4 °C. The solution was then centrifuged at 4500 RCF, 4 °C for 30 min, before 80% ammonium sulphate saturation (~43% *w*/*v*) was added and the solution placed on a magnetic stirrer at 4 °C and left overnight (>12 h). The protein-containing precipitate was then centrifuged at 4500 RCF, 4 °C for 30 min. The supernatant was removed, and the protein precipitate brought up in purified water (ratio of 15 parts water to 1 part supernatant), vortexed for 2 min, followed by centrifugation at 14,000 RCF, 4 °C for 30 min. Subsequently, the supernatant was diluted 1:9 with cold 1:4 acetone:methanol. The solution was placed at −20 °C and allowed to precipitate overnight (>12 h). The solution was subsequently centrifuged at 14,000 RCF, 4 °C for 30 min, and the supernatant was discarded. The pellet was left to evaporate at room temperature for 1 h, then resolubilised in purified water. The total protein concentration was then measured using a Thermo Scientific Nanodrop 2000 Spectrophotometer in A280 mode (Wilmington, DE, USA). Extracted venom proteins were stored at −80 °C. 

### 4.2. Cardiovascular Assays

The effect of venom on blood pressure and heart rate was determined using the previously described anesthetised rat preparation [34]. This procedure was approved by the Monash Animal Research Platform (MARP) Animal Ethics Committee, Monash University, Australia MARP/2014/97 (approved in December 2014). 

### 4.3. Neurotoxicity Assays

The *in vitro* neurotoxicity of venoms was tested using the chick biventer cervices nerve-muscle preparation as described previously [35,36]. This procedure was approved by the Monash Animal Research Platform (MARP) Animal Ethics Committee, Monash University, Australia MARP/2014/97 (approved in December 2014). 

## Figures and Tables

**Table 1:** T_50_ values for fish venoms at 1 µg/mL (mean ± SEM).

Species	t_50_ (min)
*N. kuhlii*	19 ± 0.3
*H. toshi*	7 ± 0.1 *
*P. fuscus*	22 ± 0.2

* Significantly different compared to *N. kuhlii* and *P. fuscus* (*p* < 0.05) one-way ANOVA; *N* = 4.
